# Parental levels of stress managing a child diagnosed with type 1 diabetes in Riyadh: a cross sectional study

**DOI:** 10.1186/s12888-019-2414-y

**Published:** 2020-01-03

**Authors:** Mohammed Aldubayee, Salaad Mohamud, Khaled Ayman Almadani, Abdullah Abdulrahman Alabbad, Abdulaziz Ghazi Alotaibi, Abdulhakim Ali Alkhodair, Amir Babiker

**Affiliations:** 1 Department of Pediatrics, King Abdullah Specialized Children’s Hospital (KASCH), King Abdulaziz Medical City (KAMC) , Ministry of National Guard-Health Affairs (MNGHA), Riyadh, Saudi Arabia; 2 King Saud bin Abdulaziz University for Health Sciences (KSAUHS), Ministry of National Guard-Health Affairs (MNGHA), Riyadh, Saudi Arabia; 3 King Abdullah International Medical Research Center (KAIMRC), Ministry of National Guard-Health Affairs (MNGHA), Riyadh, Saudi Arabia

**Keywords:** Children, Diabetes, Parents, Riyadh, Saudi Arabia, Stress, T1D

## Abstract

**Background:**

Caring for a child with Type 1 Diabetes (T1D) pose a significant burden on parents especially when they struggle with their child’s T1D management. The experience of not coping or struggling to cope increases the level of stress in parents, which may adversely affect their child’s diabetic control (Al Dubayee et al, Horm Res Paediatr 88:2019). In this study, we assessed the level of stress parents experience in caring for a child diagnosed with T1D in four different domains.

**Methods:**

This was a cross-sectional study conducted in two specialized diabetic centers in Riyadh, Saudi Arabia, from February to May 2015 (Al Dubayee et al, Horm Res Paediatr 88:2019). We used an Arabic translation of the validated Pediatric Inventory for Parents (PIP) questionnaire. The frequency and perceived difficulty of stressful events were rated by interviewing parents caring for children with T1D using two 5-point Likert scales.

**Results:**

The sample realized as 390 parents. The level of stress increased in separated and unemployed parents. The frequency (mean 64.9/210, SD 7.529) and difficulty (mean 65.3/210, SD 9.448) indices of the parental level of stress were compared with variables possibly associated with stress. Both of the frequency difficulty indices correlated with the marital status, the father’s level of education and occupation as well as HbA1c level (*P*-value < 0.05). In addition, the frequency index correlated with the frequency of hypoglycemia and the difficulty index correlated with the number of children in the family (*P*-value < 0.05).

**Conclusion:**

Parents of children with T1D in Riyadh experience a significant level of stress that may affect the child’s glycemic control (Al Dubayee et al, Horm Res Paediatr 88:2019). Assessing the level of stress and providing support for these families has the potential to improve the clinical outcome.

## Background

Type 1 diabetes (T1D) is a chronic illness characterized by insulin deficiency, which occurs as a consequence of the progressive destruction of the beta cells in the pancreas [1]. Frequent symptoms are increased thirst, frequent urination, fatigue, weight loss, and if the blood glucose level is high, diabetic ketoacidosis [2]. Internationally, T1D affects approximately 1.7 per 1000 persons under the age of 20 years, and the rate is increasing with 2–5% annually [3]. In Saudi Arabia, the prevalence of T1D in children and adolescents is 1.095 per 1000 [4]. Managing T1D requires frequent monitoring to maintain an optimum blood glucose level by either insulin injections or insulin pumps [5]. These management strategies can create a sense of burden in the parents’ minds, causing stress and anxiety. Parents are also concerned about diabetic complications, short- and long-term, as well as the expected outcome. An additional stressor is the lack of awareness regarding T1D and the treatment in the school environment [6].

Literature reports indicate that not every child with T1D received treatment at school and those who did experienced difficulties with the treatment especially with injecting insulin, which is more difficult to manage in a school setting [7–9]. The situation increased the stress in the family since the child spends most of the day at school. Another study, investigating the parental perspective, indicated that 85% of the family’s life was affected by the disease and in 44%, one of the parents had to modify their work schedule to attend the child’s needs at school [8]. Sixteen percent of the parents thought that they did not have adequate knowledge regarding the disease. When asked, the majority thought that their child was capable of managing T1D by self-injecting; however, the presence of a supervising nurse or a teacher would improve the outcome [8]. Hilliard et al. reported moderate stress levels in parents caring for a child with T1D, which was mainly due to the child misbehaving and affecting the glycated hemoglobin (HbA1c) [10]. We did not explore schooling as an important factor contributing to parental stress levels in the current study.

The aim of this study is to evaluate the degree of parental involvement in the routine care of their child with T1D in Riyadh and to assess the level of stress in parents caused by the different components of diabetic care.

## Methods

The chosen design was a cross-sectional study conducted at two centers, one at King Abdullah Specialized Children’s Hospital, Riyadh, Saudi Arabia and the Diabetes Center at King Salman Hospital from February to May 2015. A pre-validated Pediatric Inventory for Parents (PIP) questionnaire was completed by one of the authors during an interview with one of the parents of the child with T1D. The focus was the parental involvement in the management as well as the level of stress associated with caring for a diabetic child [11]. PIP comprises of four domains, including communication, emotional distress, medical care, and role function. Prior to the administration of the questionnaire, the instrument was translated into Arabic using internationally accepted forward and backward translation guidelines.

All pediatric patients with T1D and consenting parents were included. Each of the two enrollment centers has approximately 400–600 children with T1D at the time of the study with 5 diabetic clinics in both centers per week. On average, 15 pediatric patients attended every week in each clinic. We assumed 50% would be eligible to participate in the study. The calculated sample size to power this study at 85% with a significant *P*-value of 0.05 was 375 participants. We targeted at least 450 participants allowing for a 25% refusal rate.

The demographic data of the T1D patient was obtained from the electronic hospital records, including the parents’ contact information and the relevant medical information. The inclusion criteria were Saudi T1D pediatric patients (< 14 years) registered in the hospital database, diagnosed for at least 1 year, at least one parent available to be interviewed, regardless of gender. Parents, who were diagnosed with a mental illness or not primarily responsible for the care of the child with T1D, were excluded from the study.

Participants were conveniently recruited and asked to respond to questions related to 42 stressful events associated with parenting a child with T1D. The PIP consists of two components, each graded on a 5-point Likert scale, the frequency index (ranging from ‘never’ to ‘very often’) and the difficulty index (ranging from ‘not at all’ to ‘extremely’) of individual events. The internal consistency was excellent for both scales (frequency: α = 0.94, difficulty: α = 0.96).

### Statistical methods

Quantitative variables are expressed as mean and SD and categorical values as frequency and percentage. Data is presented in graphs and tables to improve the presentation. A Student t-test was used for quantitative variables and a Fisher Exact Test for the categorical data. Data was entered in a Microsoft Excel spreadsheet and analyzed using SPSS 20. A *P*-value of less than 0.05 was regarded as significant.

## Results

**Fig. 1:**
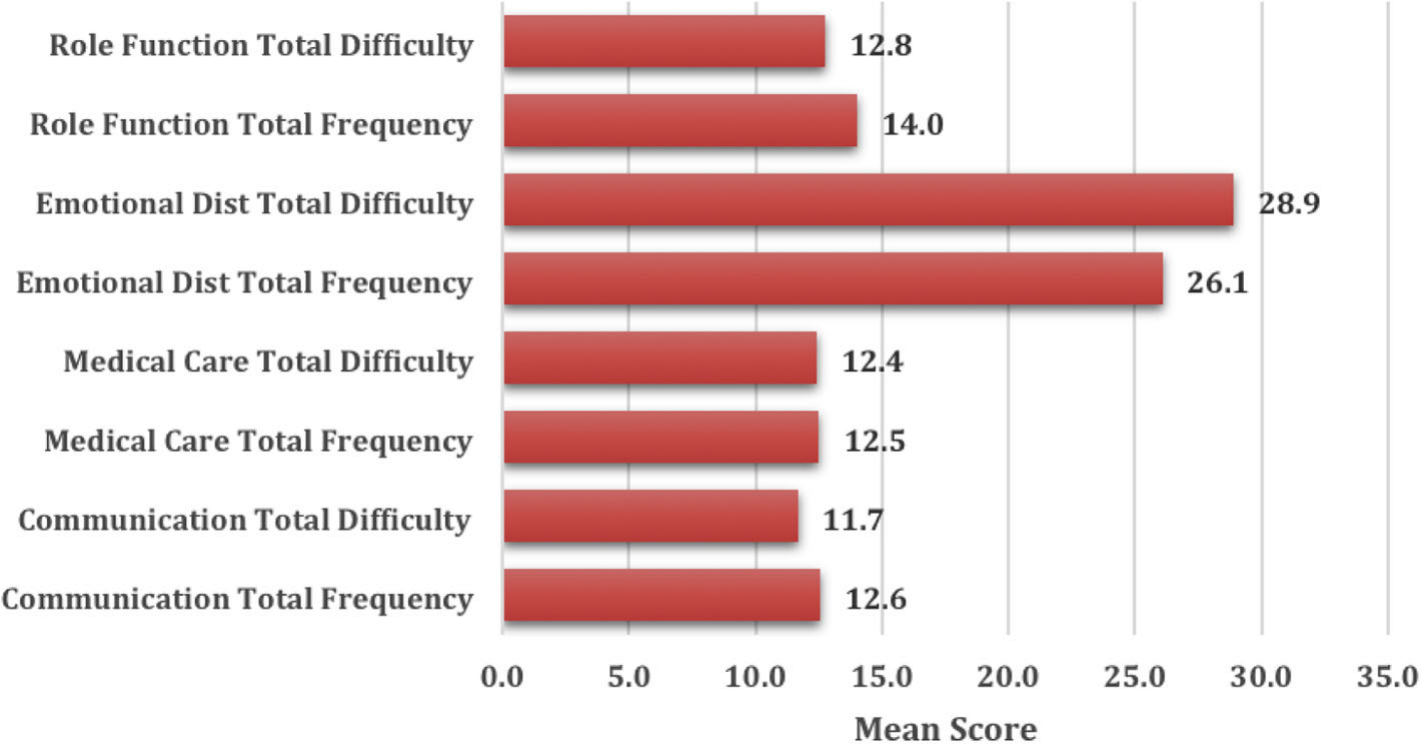
Frequency and difficulty scores of stress in parents using Pediatric Inventory for Parents (PIP) questionnaire

We interviewed 390 parents with the majority (95%, *n* = 370) mothers. In terms of gender, the majority of the pediatric T1D patients were male (55%, *n* = 215) (Table 1). The HbA1c was available for only 371 pediatric T1D patients. The total possible score (210) of the frequency and difficulty indices was the same. The mean of the frequency index of events was 64.9/210 (±7.529) and the mean for the difficulty index of events was 65.3/210 (±9.448). The highest level of stress in the frequency and difficulty indices were in the emotional distress domain (mean 26.1 and 28.9, respectively). The highest mean in this domain was associated with the long-term impact of the disease on their child and the uncertainty related to complications that may occur in the future. Parents experienced less stress in the medical care, communication and role function domains of the PIP (mean approximately 12 in each) (Fig. 1). None of the parents had to change his or her job or experienced significant absence from work to supervise their child’s management.

**Table 1:** Demographics of parents and children with type 1 diabetes

CHILD		N	Percentage
Age	1–5 years	47	12.0%
	6–10 years	142	36.3%
	11–14 years	202	51.7%
Frequency of Hypoglycemia	More than once\week	38	9.9%
	Less than once\week	344	90.1%
Age of the patient (Mean ± SD)	9.93 ± 3.19	–	–
Time since diagnosis (Mean ± SD)	3.75 ± 2.27	–	–
Last blood glucose (Mean ± SD)	200.54 ± 87.59	–	–
HBA1C (Mean ± SD)	10.13 ± 1.88	–	–
PARENTS		N	Percentage
Does the parent live in Riyadh	Yes	367	93.6%
	No	25	6.4%
Mother’s level of education	None	40	10.2%
	Elementary	104	26.5%
	Secondary	186	47.4%
	Collage	62	15.8%
Father’s level of education	None	12	3.1%
	Elementary	53	13.5%
	Secondary	171	43.6%
	Collage	156	39.8%
Marital status	Married	363	92.6%
	Unmarried	29	7.4%
Mother’s Occupation	Government Employee	58	14.8%
	None	335	85.2%
Father’s Occupation	Businessman	64	16.3%
	Dead	15	3.8%
	Government Employee	248	63.3%
	Military	20	5.1%
	None	3	0.8%
	Private Sector	11	2.8%
	Retired	31	7.9%
Number of children (Mean ± SD)	5.1 ± 2.3	–	–

*N* Number, *HBA1C* Hemoglobin *A1C*, *SD* Standard deviation

We compared the means of the frequency and difficulty indices with variables, which may be association with the level of stress in the parents, including the frequency of hypoglycemia, whether the parents lived in Riyadh (where medical care is provided), parental level of education, marital status, parental occupation and years since the diagnosis. Spearman’s correlations with frequency and difficulty scores were calculated for the continuous variables such as HbA1c and number and age of children in the study. The variables found to be associated with a higher frequency index were frequency of hypoglycemia, father’s level of education, occupation and marital status (*P*-value < 0.05) (Table 2). With regards to the level of difficulty experienced by parents, that was significantly associated with marital status, number of children in the family, both parents’ level of education as independent factors and the father’s occupation (*P*-value < 0.05) (Table 2).

**Table 2:** Mean comparisons of variables with the total frequency and total difficulty scores of level of stress in parents

		Total Frequency			Total Difficulty		
		Mean	SD	*P*-value	Mean	SD	*P*-value
Age of the patient							
1–5 yrs		68.6	10.8	0.001	69.6	10.4	0.001
6–10 yrs		65.1	6.2		65.6	8.8	
11–14 yrs		63.8	7.0		64.0	9.0	
Insulin regimen							
Conventional		65.0	7.5	0.035	65.5	9.4	0.036
Intensive insulin therapy		57.0	8.3		55.5	7.1	
Frequency of Hypoglycemia	More than once\week	67.8	12.3	0.018	67.9	11.7	0.094
	Less than once\week	64.5	6.8		65	9.1	
Does the parents live in Riyadh	Yes	65	7.6	0.516	65.4	9.4	0.607
	No	64	6.9		64.4	10	
Mother’s level of education	None	66.4	8.4	0.375	69.1	9.9	0.002
	Elementary	65.4	7.3		66.9	10.4	
	Secondary	64.3	6.4		63.6	7.9	
	Collage	65	10		65.6	10.8	
Father’s level of education	None	63.5	6.3	0.005	66.6	8.1	< 0.001
	Elementary	67.5	8.3		69	9.6	
	Secondary	65.5	7.3		66.3	9.7	
	Collage	63.5	7.3		63	8.7	
Marital status	Married	64.5	7.2	< 0.001	64.9	9.1	0.001
	Unmarried	70.6	9.9		71.3	12.1	
Mother’s Occupation	Government Employee	64.8	10.6	0.904	64.6	11.8	0.562
	None	64.9	6.9		65.5	9	
Father’s Occupation	Businessman	65.6	7.6	< 0.001	66.7	9.2	< 0.001
	Dead	67.7	5.5		67.7	8.6	
	Government Employee	64.1	7.2		63.9	8.7	
	Military	68.1	7.8		67.5	8.5	
	None	82	8.9		90.3	11.8	
	Private Sector	70	9.3		71.1	11.8	
	Retired	63.5	6.4		67	10.6	
Years since diagnosis	1	65.7	7	0.134	65.1	8.9	0.339
	2	66.5	10.2		66.8	11.4	
	3	65.6	6.6		66.4	8.6	
	4	63.5	5.6		63.9	7	
	5	63.9	6.8		65.5	9.7	
	6+	63.9	7.4		63.9	9.4	

The level of stress was high for separated parents; however, being employed had a lower level of stress. For the continuous variables, the HbA1c level and age of the child correlated with the level of parental stress and the last blood glucose level correlated with the level of difficulty experienced by the parents (Table 3).

**Table 3:** Spearman’s correlations of continuous variables with the total frequency and total difficulty scores

Variables	Spearman’s correlation with Total Frequency score	Spearman’s correlation with Total Difficulty score
Age of the children	−0.171**	−0.201**
HBA1C	0.279**	0.379**
Last blood glucose	0.095	0.207**
Number of children	0.001	0.063

** Significant correlations (*P* value< 0.01)

## Discussion

T1D is a life-long condition resulting in major health and psychosocial complications for the child, their families and the whole community. Diabetes management involves insulin injections, dietary control and adjustment of doses in relation to exercise and insulin sensitivity during the day. Poor management and non-adherence to strict regimens are persistent problems in children. Parenting and Parent-child interactions were identified as crucial points of intervention to support such families to achieve emotional wel-being and better glycemic control. However, only a few parenting interventions have been developed or evaluated for parents of young children [12].

Regarding the glycemic profile, Hilliard et al. found that high levels of family conflict and stress were related to poor glycemic control [10]. Their results suggested that the parental stress level was moderate and primarily due to the child’s misbehavior, which may have affected the glycemic control. It was also reported that the higher the involvement of the family, the better the outcome of disease management [13]. The mothers’ knowledge and socioeconomic status had a major influence on glycemic control in T1D pediatric patients [14]. Several studies indicated that the mother is usually responsible for most of the diabetic care of the child, resulting in the father experiencing a lower level of anxiety and fear of hypoglycemic attacks [15–18]. In the current study, variables associated with a high level of stress were the frequency of hypoglycemia, the father’s level of education and occupation, marital status and the HbA1c level. Support for some of the variables, the frequency of hypoglycemia, marital status and HbA1C, is found in literature [17–21]. The mean of the frequency and difficulty indices in the current study is lower than reported in a similar study in the United States (102.36 and 93.36, respectively) [12]. We speculate that local factors such as cultural differences and the impact of religion on spirituality, social acceptance of the disease, and family support may play a role.

Our study focused on four major domains, including communication, emotional distress, medical care, and role function. The communication domain consisted of 9 items such as “Arguing with family members”, “Speaking with your child” and “Talking with the doctor”. “Speaking with the child” scored the highest in the frequency index. Clinicians could emphasize this issue by encouraging the parents to adopt new tactics to communicate with the child as communication with children can be challenging. Communication can be improved by spending an appropriate time with the parents during the clinic visits and using simple language especially when explaining specific procedures. The emotional distress domain included 15 items. Some of these items are related to observing their children going through different scenarios such as “Knowing that their child is hurt”, “Seeing their child sad”, and “Thinking about their child being isolated due to his/her disease”. Other items are related to the parents themselves, such as “Waiting for test results”, “Experiencing sleep difficulties”, and “Learning about bad news”. To manage emotional distress, parents should attend educational programs and become knowledgeable about their child’s disease, which will reduce the level of stress. The third domain is medical care with 7 items. This domain focuses on the parents’ perceptions of medical care such as bringing the child to the clinic as well as watching or handling procedures such blood glucose monitoring and insulin injections. The last domain is role function, with 10 items. This domain explored the living restrictions that parents may experience. In the current study, parental job attendance was not affected by their child’s illness compared to 44% of the parents in another study [17].

Although the difficulty index is more informative in reflecting the level of stress, the frequency index can vary due to age and in different families due to psychosocial dynamics, however, if interpreted in conjunction with the difficulty index, it adds valuable insight [22]. As previously described in literature, other important factors that are significantly associated with the difficulty experienced by parents in the current study were the marital status, number of children in the family, and the parental level of education [11, 22].

## Conclusion

Parents of children with T1D in Riyadh experience a considerable level of stress, correlating with the child’s glycemic profile that could affect the diabetes control. The parental level of stress should be periodically assessed, and to provide optimum care, psychosocial support should be incorporated as part of routine care for these patients and their families.

## Data Availability

The datasets used and/or analyzed during the current study available from the corresponding author on reasonable request.

## Abbreviations

HbA1c: Hemoglobin A1c
KSAU-HS: King Saud Bin Abdulaziz University for Health Sciences
PIP: Pediatric Inventory for Parents
T1D: Type 1 diabetes
